# Advancements in additive manufacturing for video laryngoscopes: a comprehensive scoping and technological review

**DOI:** 10.1186/s13643-023-02406-y

**Published:** 2023-12-14

**Authors:** Ana Cristina Beitia Kraemer Moraes, Chiara das Dores do Nascimento, Everton Granemann Souza, Mauricio Beitia Kraemer, Mauricio Moraes, Neftali Lenin Villarreal Carreno, Evandro Piva, Rafael Guerra Lund

**Affiliations:** 1 Pelotas Dental School, Graduate Program in Dentistry, Federal University of Pelotas, Pelotas, RS 96010-560 Brazil; 2 Master’s Degree in Electronic and Computer Engineering, Center for Social and Technological Sciences, Catholic University of Pelotas, Pelotas, RS 96015-560 Brazil; 3 Faculty of Medicine, São Francisco University, Bragança Paulista, SP 12916-900 Brazil; 4 Faculty of Medicine, Federal University of Pelotas, Pelotas, RS 96010-560 Brazil; 5 Graduate Program in Materials Science and Engineering, Technological Development Center, Federal University of Pelotas, Pelotas, RS 96010-610 Brazil

**Keywords:** Intubation equipment, Video laryngoscopes, Additive manufacturing, Fused deposition modeling, Technological aspects

## Abstract

The global healthcare crisis with the COVID-19 pandemic has placed a significant overwhelming demand for intubation procedures and the need for reliable and accessible video laryngoscopes. The purpose of this scoping and technological review is to provide a comprehensive overview of the current state of the art, covering the period from 2007 to 2022, pertaining to the manufacturing process, characteristics, and validation of video laryngoscopes produced using additive manufacturing techniques. Following the guidelines outlined in the Preferred Reporting Items for Systematic Reviews and Meta-Analyses extension for Scoping Reviews (PRISMA-ScR), an exhaustive search was conducted across nine prominent databases (PubMed, Web of Science, Scopus, Cochrane, Prospero, Scielo, Embase, Lilacs, Virtual Health Libraries-VHL) and four patent databases (EPO/ESPACENET, WIPO/PATENTSCOPE, National Institute of Industrial Property (INPI), Google Patents). The main materials utilized for the impression, as well as the physical characteristics of the device are introduced at first. Crucial aspects to facilitate proper visualization of the anatomical structures during endotracheal intubation as the optimal angulation of the blade, the mechanical resistance of the device, traction force on the jaw, intubation time, and the experimental methods employed to validate its performance were reviewed in terms of their recent advances.

**Supplementary Information:**

The online version contains supplementary material available at 10.1186/s13643-023-02406-y.

## Introduction

The global healthcare crisis with the COVID-19 pandemic evidenced the overwhelming demand for intubation procedures and the need for a video laryngoscope has become more pronounced. Additive manufacturing has emerged as a promising solution, enabling the rapid production and prototyping of video laryngoscopes [1, 2].

Fused deposition modeling (FDM) has been widely employed since 2013 [3, 4], and polymers and filament have been explored to introduce the manufactured video laryngoscopes. Acrylonitrile butadiene styrene (ABS) and polylactic acid (PLA) are the predominant polymer materials utilized for prototyping, and their use is indicated in airway access training or for the projection of a new device [5–7]. When the demand calls for a more resistant material, exceeding 400 intubations, the primary choice is polycarbonate, utilizing electronic injection, as opposed to PLA, with an average number of 100 intubations [8].

The video laryngoscope blade is an essential part of the device. With a continuous curve in its design, initially described by Robert Macintosh in 1943 [9, 10], it can influence the performance and success of intubation [11]. Studies have shown that hyper-angulated blades perform better. Some studies indicate that blades with an angle of 70° allowed 100% success in orotracheal intubation, compared to 89% success with 90° blades [7, 11]. The blade angulation is also related to the traction force used for jaw opening and the laryngeal visualization time, providing ergonomic characteristics and resistance to equipment use [7]. The study of forces exerted on the handle and blade is essential to assess the lifespan of these blades and to evaluate the rate of complications with patients.

The identified traction forces vary among the different devices, primarily depending on the design and type of material used in the construction of the equipment. However, these data are scarce, as authors do not always prioritize this type of assessment in their studies, which are mostly clinical studies, often neglecting the detailed analysis of the device's design and mechanics [12]. Thus, a compilation of this data can assist in optimizing future devices.

Regarding the validation process of manufactured devices, a series of experiments and tests are conducted to assess their performance and functionality. This includes the analysis of factors such as ease of use, maneuverability, intubation success rate, time required for intubation, and potential complications during the procedure [7, 9]. Additionally, validation studies may involve comparisons between video laryngoscopes and other existing devices or techniques, simulated intubations on mannequins or cadavers, and evaluation of their efficacy in clinical settings with real patients. Although the use of mannequins for validation is not considered ideal, it is important because it allows for the assessment of the device in a standardized difficult airway scenario and its functionality before being validated in patients (Table 1) [10].

**Table 1:** Description of randomized clinical trials that compared 3D printing prototypes with commercial equipment in mannequins

Author/year	*N*	Comarck-Lehane identification	Laryngeal visualization time	Intubation time	Intubation success rate	Intervention/control
Cohen T; Nishioka H./2017 [13]	64 (anesthesiologists)	VLB:100% MAC: 21% (*p* = 0.000)	VLB:16.6seg MAC:39.1seg (*p* = 0.001)	VLB:55.4seg MAC:91.8seg (*p* = 0.042)	VLB:94.1% MAC:60% (*p* = 0.003)	VBL (3D) MAC blade (commercial)
Lambert C; John S; John A./2020 [7]	43 (professionals)	Pentax vs TVL vs Macintosh (*p* < 0.001)	No	TVL 17.5seg Pentax15.5seg Macintosh 27seg (*p* < 0.0001)	TVL 88% Pentax 97.7% Macintosh 67.4%	TVL (Tanser)(3D) Pentax AWS(3D) Macintosh (commercial)
De Villiers C; Alphonsus C; Eave D; et al. 2021 [8]	100 (experienced anesthesiologists and consultants)	No	No	VLP(3D):13.3 s MAC:18.2seg	No	VLP(3D) CMAC (commercial)
Ataman A; Altina E 2021 [1]	23 (emergency physicians and clinicians > 2 years of experience)	No	AirAngel 13.6seg Glidescope 8.1seg	AirAngel 27.7seg Glidescope 20.1 seg	AirAngel 56% Gladescope 87%	AirAngel (3D) Glidescope (commercial)
Fonternel T; Rooyen H; Joubert G; Turton E	36 anesthetics	C-MAC 80.6% class 1 Novel Device 50% (*p* = 0.0045)	C-MAC 5seg Novel Device 9.4seg (*p* < 0.001) (CI = 6.2–1.0)	C-MAC 13.8seg Novel Device 19seg (*p* = 0.001)	100%	C-MAC^R^ VL with D-blade

Considering these factors, we provide a comprehensive overview of the characteristics and protocol validation of the 3D-printed equipment.

The manuscript begins with a detailed account of the search strategies, descriptors used, databases employed, and eligibility criteria. Next, a flowchart illustrates the methods of study identification, screening, and inclusion. The results section provides an overview of the historical context and major trends in additive manufacturing for video laryngoscopes (VLPs). Additionally, aspects related to filament selection, forces applied for oral cavity opening, and equipment strength are discussed in this section. Furthermore, the literature’s suggested angulation values, equipment validation mechanisms, and industrial applicability are addressed. Finally, the discussion and conclusion sections offer our perspectives on future research directions. Authors are encouraged to follow this structure to effectively present their findings and insights.

## Methods

### Search strategy

This scoping review adhered to the recommendations and checklist derived from the Preferred Reporting Items for Systematic Reviews and Meta-Analyses extension for Scoping Reviews (PRISMA-ScR) [14, 15]. The study search and selection process started in December 2021 and concluded in October 2023. No search restrictions were applied based on the year of publication, language, or study type. A comprehensive search was conducted across various electronic databases, including PubMed, Web of Science, Scopus, Google Scholar, Cochrane, Prospero, Scielo, Embase, Lilacs/Bireme (VHL), and medRxiv, as well as Portal CAPES–BDTD (Biblioteca Digital Brasileira de Teses e Dissertações), without applying any filters or restrictions to the studies. Additionally, technological documents were sourced from EPO/ESPACENET, WIPO/PATENTSCOPE, and the National Institute of Industrial Property (INPI). Gray literature from Google Scholar and Google Patents was also consulted.

### Descriptors and databases

The survey in databases employed Boolean operators “OR” and “AND” to identify relevant descriptors from MeSH, Emtree terms, and DeCS. The search terms included (Laryngoscopies) OR (Laryngoscopes) OR (Laryngoscope) OR (Laryngoscopy) OR (Intratracheal Intubation) OR (Endotracheal Intubation) OR (Intubation) OR (Laringoscopio) AND (Three-Dimensional Printing) OR (3-Dimensional Printing) OR (3 D Printing). Another combination was employed for the Portuguese and Spanish databases, Scielo and Lilacs/Bireme (VHL), using terms (Laringoscopia) OR (Intubação) OR (Intubação Endotraqueal) OR (Intubação intratraqueal) AND (Manufatura aditiva) OR (3D Impressão).

Additionally, a patent search was conducted using the International Patent Classification (IPC) approach with codes A61 (medical or veterinary science), A61B (diagnosis, surgery, identification), and the groups and subgroups A61B1/267 (laryngoscopes), and A61B1/05 (camera in the distal end portion). A combined approach was utilized, employing IPC with the terms (Laryngoscope) OR (Laryngoscopy) AND (3D Printing) OR (3-Dimensional Printing) OR (Three-Dimensional Printing), (Laringoscopia) OR (Intubação) OR (Intubação Endotraqueal) OR (Intubação intratraqueal) AND (Manufatura aditiva) OR (3D Impressão).

### Eligibility criteria

The eligibility criteria or inclusion in this review encompassed the manufacturing process of video laryngoscope equipment or its components using additive manufacturing techniques, either with or without the integration of micro-cameras or a borescope.

Additionally, descriptive, and comparative studies examine the differences between commercially available video laryngoscopes and those produced through additive manufacturing were included, along with clinical trials utilizing mannequins for comparison and usability assessments.

Articles solely focusing on the additive manufacturing of accessories, such as the blade, system function for commercial video laryngoscopes, traditional commercial laryngoscopes, or those intended for animal use, were excluded from consideration. No restrictions were imposed on publication dates or languages, and relevant articles and technological documents considered were translated into Portuguese for analysis (Supplementary Table S2).

### Extraction strategy

A two-stage screening process was implemented to assess the relevance of studies identified in the search, involving three reviewers at different stages. The sequence for identifying and selecting documents was as follows:

- Identification of scientific studies, conference proceedings, and existing theses.
- Initial title-based selection, discarding those lacking the keywords.
- Review of abstracts to identify the subject and its relevance to the research, excluding irrelevant ones.
- Full article review.
- Selection of relevant articles for the study.

In the initial stage, the title alone was considered as a search criterion by two reviewers, and the Mendeley Reference Manager was employed to add selected articles based on the identification of eligibility criteria and the removal of duplicates. In the second stage, abstracts were reviewed, by the same two reviewers, and studies that did not meet the eligibility criteria were excluded.

The assessment of patents involves an evaluation of their titles, abstracts, claims, and drawings. A third reviewer examined the patent publications in the patent database. Furthermore, additional criteria, including the International Patent Classification (IPC) code, publication date, international registration (PCT), participation of international entities with technological significance, and commercial potential, were taken into account to determine their relevance. The sequence for identifying and selecting documents proceeded as follows:

- Title selection.
- Review of patent abstracts and IPC classification.
- Review of the invention description.
- Review of claims and verification of drawings.
- Industrial applicability for those with WIPO (PCT) patents.

## Results

### Study selection

An electronic search was conducted in December 2021 and October 2023 resulting in an initial retrieval of 1588 publications. After removing duplicate entries and screening for relevance based on the title, 1507 publications were excluded as they did not meet the eligibility criterialeaving 86 studies for abstract evaluation. Further evaluation of the full text led to the exclusion of 59 studies, resulting in 22 studies met the inclusion criteria. In the patent database, the search retrieved 2502 patents. After reviewing the titles, abstracts, and drawings, 2484 were excluded.

**Fig. 1:**
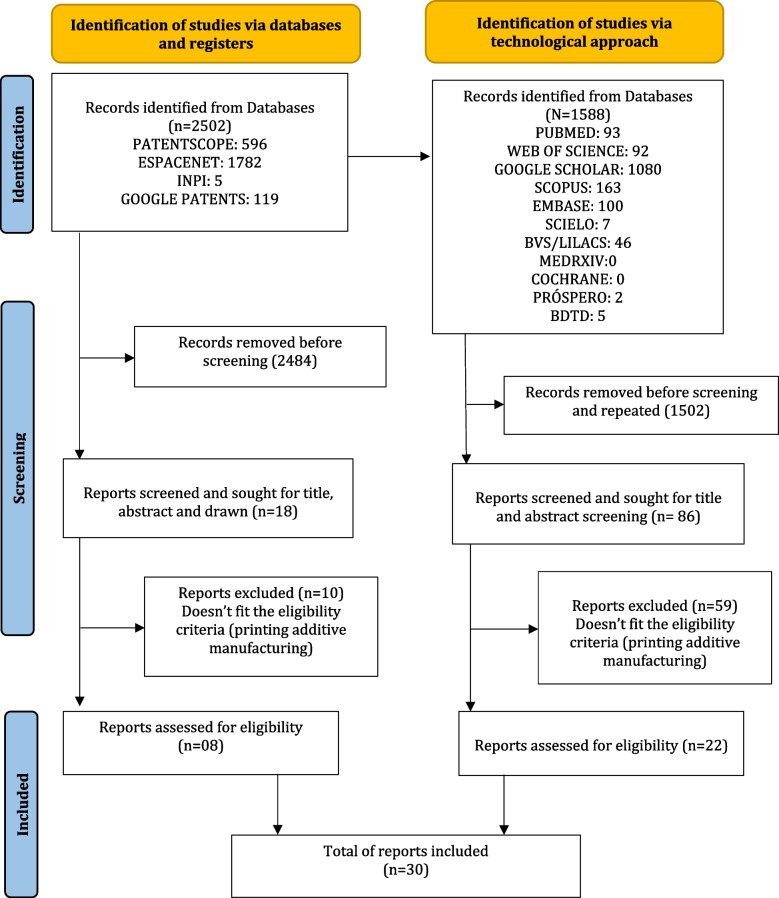
PRISMA flowchart of the included studies and technological approach

Among the remaining 18 patents, 10 were excluded based on the exclusion criteria, leaving 8 patent documents for analysis. Overall, 30 documents, including both studies and patents, were considered relevant to the search. Figure 1 presents a PRISMA flowchart that provides a visual summary of the articles, from the initial identification stage to the final inclusion stage.

### Study characteristics

The search retrieved scientific articles dating back to 2007; however, only publications from 2016 onwards met the selection criteria, as did the patents. Of the selected articles, 15 mentioned the presence of a camera or endoscope, while the remaining 7 did not reference this accessory. In the technological database search, 8 patents mentioned the presence of an endoscope or microcamera. A technological approach identified a total of 19 publications, five originating from the USA and the remaining publications from emerging countries, including Brazil (2), Argentina (1), Equator (1), México (1), South Africa (2), and Turkey (1). Among the identified publications, the majority consisted of scientific articles (six in total), followed by four clinical randomized studies and one systematic review. The demographic data of the included articles can be found in Supplemental Table S1. Regarding patents, a selection process yielded a total of 10 relevant publications. The patent applications originated from various countries, including Brazil (4), Chile (2), Greece (1), Spain (1), China (1), and the United Kingdom (1).

### Trends in VLP in additive manufacturing

The trends in video laryngoscope (VLP) development through additive manufacturing have emerged relatively recently. Early publications primarily focused on the initial stages of producing laryngoscope prototypes for otolaryngology, utilizing processes like light-curing technology [16]. Notably, patents spanning from 2007 to 2021 have extensively employed ABS photopolymerization and fused deposition techniques for VLP fabrication. Fused deposition modeling (FDM) has emerged as a prominent choice for rapid prototyping of laryngoscopes, reflecting a prevailing trend in the field [2, 17]. This evolution in additive manufacturing techniques has ushered in new pathways for enhancing VLP design and manufacturing methods, aligning with the evolving needs of the medical field, and advancing the potential for improved patient care.

### Filaments of choice

The utilization of fused deposition modeling (FDM) for impressions, particularly with the thermoplastic polymer ABS, has been highlighted as the favored approach for rapid prototyping. However, it is essential to note that ABS equipment should be confined to training exercises using simulators due to its carcinogenic properties and lack of compatibility with in vivo tissue [2, 17, 18].

Since the advent of the COVID-19 pandemic in 2020, there has been a heightened emphasis on adopting environmentally friendly thermoplastic polymers. Polylactic acid (PLA) has emerged as a biocompatible, biodegradable thermoplastic polyester sourced from renewable materials that pose no harm to human health [6, 19]. Furthermore, augmenting PLA with materials like carbon or onyx has exhibited enhanced physical attributes, filament robustness, and product longevity [20, 21]. While the incorporation of PLA with other filaments enhances polymer properties, it may also escalate the cost of prototypes, which are still in the exploratory study phase [2, 17, 21].

Recent explorations have delved into novel combinations of thermoplastic polymers, including polyethylene terephthalate glycol (PETG) [21], CCTREE polypropylene [8], and Nylon [8]. Technological resources have also noted alternative materials such as quartz, silica, sustainable vegetable plastic, ASA, Tristan, and Nylon, all of which bestow durability on the device.

This evolving landscape of thermoplastic material investigations provides a foundation for enhancing the development of video laryngoscopes (VLP) through additive manufacturing, aligning with the ongoing progression of medical requirements, and elevating the potential for advancing patient care.

### Design process

In the development of the 3D video laryngoscope, Londoño et al. [22] employed the BioDesign Innovation Process methodology, a multidisciplinary approach for medical equipment development projects. The method initially involves exploring the problem that led to the need for the product's creation. Subsequently, the device is designed using computer-aided design software, including engineering and modeling software such as Solid Works and AutoDesk Mesh Mixer [7, 22]. The prototype is then manufactured using 3D printing/FDM with various filaments. Finally, the device is tested and validated [22].

In the initial design process, the model must possess certain features, as identified by Huysamen [20]: it should accommodate a microcamera passing through the blade; the blade should have an adjustment to prevent camera rotation, aiding in the correct procedure orientation. The device should allow the passage of light and the tube. If the blade is attached to the handle, i.e., not inserted, it should be as ergonomic as possible to avoid interfering with intubation [21]. According to Lambert et al., the model should prioritize safety, effectiveness, low cost, and reusability [7].

Other characteristics relevant to the device design were described, including a hyper-angulated blade similar to those of the Airtraq and Pentax WS (Pentax-Airway Scope; AWS-S10; Tokyo, Japan), as well as increased blade thickness for patients with limited mouth opening [7].

### Related forces

Although sparsely discussed, several considerations regarding the resistance and force exerted on a video laryngoscope can be inferred from the literature references. Generally, two types of forces are assessed in studies: the force required to open the oral cavity and the equipment's resistance force, associated with a minimum usage force. This latter force is relevant due to the potential for deformities and fractures in the device.

References [23] and [21] report force values capable of inducing deformity alterations in the device. In both studies, device resistance was linked to the filament material employed in video laryngoscope fabrication. Specifically, in [21], the material polyethylene terephthalate glycol-modified (PETG), renowned for its robustness and ease of printing, was chosen. Finite element analysis (FEA) was employed to assess stress distribution. Results revealed that the ideal blade resistance was achieved at a force of 100N, characterized by a mass of 102 g, Von Mises stress of 29.0 MPa, and volume of 79 cm^3^, suggesting the viability of using PETG filament for the video laryngoscope blade. Other researchers, as in [23], studied ABS and identified a minimum force of 84N as the required resistance for safe blade usage.

Regarding the maximum force necessary for opening the oral cavity, a technical document [13] compared, through experimental trials mannequins, an ABS-manufactured device with a metal-blade Macintosh laryngoscope. The authors obtained an average force of 18.18 N for the ABS VLP, compared to 22.87 N for the metal device. Although the devices had different masses, the authors attributed the results mainly to the optimized angulation of the ABS VLP relative to the metal one, rather than the devices’ masses themselves, which they claimed minimally influenced the direction of applied jaw-opening traction force. These findings align with the study by [11], which asserts that a commercial Gladescope video laryngoscope equipped with a Macintosh blade requires a minimum force of 25 N for adequate laryngeal visualization.

### Blade angle and hygiene

The Macintosh blade design is the most prevalent among the examined studies, primarily due to its capacity to ameliorate minor intubation difficulties and optimize neck mobility angle. Beyond these factors, further advantages have been underscored with the utilization of this blade, including ease of attachment, illumination, field of view, clinical applicability, applied force, and intubation duration, all of which were significantly influenced by the blade type [23]. Regarding blade angles, some authors emphasize that hyper-angulated blades offer the capability to achieve a comprehensive view of the glottis, even in the presence of a large tongue and restricted neck mobility, with a majority of participants able to attain a complete glottis view using video-assisted devices [7, 18]. Ideal blade angulations vary, ranging between 70° and 90° according to Moraes et al. [21], and 45° and 90° for Cohen et al. [13]. Angulations between 0° and 15° are considered challenging for usage, while those between 45° and 60° have been described to facilitate cervical hyper angulation for improved visualization [18, 23, 24].

Regarding the cleansing of metal blades, it was demonstrated that standardized disinfection techniques did not effectively neutralize proteinaceous materials present in secretions such as blood, which come into contact with the blade and handle. In a study evaluating the disinfection process of 100 conventional laryngoscopes, a notable 38% contamination rate of the handle was observed, with the presence of Streptococcus viridians in the culture, thereby questioning the efficacy of the process and suggesting the use of disposable blades [25]. The recommended hygiene procedure involves initiating the process at a temperature below 35 °C using an ultrasonic device, capable of removing proteins and preventing coagulation, low-temperature tests could be employed [21]. This is followed by rinsing with warm detergent to ensure proper removal, ultimately culminating in thermal disinfection for reusable blades [23, 26].

For devices manufactured through additive manufacturing, no specific sterilization and cleansing protocol has been indicated. Instead, standardized hospital protocols for reusable devices and ethylene oxide disinfection are suggested [21]. As of now, there are no studies addressing microbial contamination or validation of hygiene and disinfection processes for these additive manufacturing devices, only for those made of metal or industrial-grade plastic.

### Equipment validation

In our study, comparative data were obtained using metal laryngoscopes, video laryngoscopes with Macintosh blades, and 3D-printed video laryngoscopes on simulation mannequins with experienced physicians using the devices. Team training, resistance tests, and comparisons with commercial video laryngoscopes were conducted using SimMan3G simulation mannequins with ease and difficult airway, involving a fully inflated tongue and stiff neck, to assess the efficacy of the 3D-printed video laryngoscope. A total of 266 professionals experienced in intubation procedures participated in the studies [1, 7, 8, 21, 24].

Five experimental studies examined the performance of the 3D-printed video laryngoscope on mannequins [1, 7, 8, 21, 24]. Intubation success rates and intubation times were among the key parameters analyzed in these studies.

The intubation time was significantly shorter when using the 3D-printed video laryngoscope developed by De Villiers et al. [8] (average time 13.3 s, minimum time 5.1 s), compared to the devices developed by Ataman et al. [1] and Lambert et al. [7] (27.7 s and 17.5 s, respectively). Additionally, Ataman´s and Cohen’s [1, 24] models, which were developed based on the Airangel and Macintosh models, exhibited comparable and shorter laryngeal visualization times (13.6 s and 16.6 s, respectively) when compared to commercial Glidescope and Mac group/Mac cable equipment (8.1 s and 39.1 s) [3, 15, 21, 27].

Furthermore, the intubation success rate was higher for the Cohen model (94.1%) when compared to other 3D-printed prototypes and commercial devices [7, 9, 10].

While experimental tests have been conducted on mannequins, further studies need to be undertaken to evaluate the suitability of this equipment for human use. Limitations such as fog or anatomical features must be taken into account, as they cannot be properly assessed in mannequins [1].

However, a systematic review [28] of five randomized studies conducted on actual patients concluded that there is limited evidence supporting the use of 3D-printed video laryngoscopes in clinical practice. This limited evidence is attributed to the absence of standardized protocols and highlights the consideration of potential risks, such as injuries, as factors that underscore the safety of device use in real patients [28].

In the same study, it was identified that the highest rates of intubation success and intubation times, along with the lowest rates of complications, were achieved when inexperienced physicians utilized the Gladescope brand’s Macintosh-bladed video laryngoscope. On the other hand, no significant differences were observed between devices when used by experienced anesthesiologists [7, 9, 10].

### Industrial applicability

Two documents, PCT WO2019075588A1 [29] and PCT WO2020003192A1 [28] offer valuable insights into the potential and industrial applicability of video laryngoscopes. These documents suggest the fabrication of devices using durable materials such as metal, polycarbonate, and polymers. Moreover, the publication WO2015104444A1 [27], accompanied by an international preliminary report on patentability, recommends a hyper angulation of the blade within the range of 30 to 60 degrees, exhibiting characteristics akin to well-established video laryngoscope models like the PENTAX AWS, AIRTRAQ, AMBU KING VISION, MCGRATH GLIDESCOPE, C-MAC, and VIVIC-TRAC. This patent introduces promising features and functionalities comparable to those found in industry-standard equipment.

## Discussion

The objective of conducting this scoping and technological review is to present a holistic exploration of contemporary advancements. Encompassing the timeframe from 2007 to 2022, this review delves into the manufacturing processes, attributes, and validation protocols of video laryngoscopes crafted through additive manufacturing methods.

In the realm of additive manufacturing, the availability of materials accessible for widespread use in 3D printers has led to notable choices such as ABS and PLA filaments. These materials have found applications in prototyping, validation, and airway access training on mannequins [12, 17]. Nevertheless, limitations are associated with the use of ABS, primarily due to its carcinogenic characteristics and endocrine effects. On the other hand, PLA, while more prone to deformity over time, can exhibit layer delamination and bacterial accumulation upon repeated use, owing to its porous nature. Given the prevalence of 3D printing in medical devices, alternative materials have gained attention, characterized by enhanced mechanical and thermal resilience, as well as compatibility with ethylene oxide sterilization. Prominent among these materials are PETG, and PLA combinations infused with elements like carbon, silica, and onyx, along with PC-ISO (polycarbonate) [21]. However, the paramount consideration resides in the identification of filaments deemed safe for human contact, adhering to the regulations set forth by North American health agencies, exemplified by Rokit’s plastics and Skinflex [18].

The design methodology has proven to be relevant, although it is not extensively described in the studies and patents. It should be noted that blade characteristics are crucial in optimizing laryngeal access in the device design [7]. Regarding the feasibility of employing certain materials in the design of a video laryngoscope, tests evaluating force to appraise material strength and force required for laryngeal visualization have been expounded upon [12]. In ABS devices, greater resistance to force-induced motions was noted [13]. Studies involving combinations of thermoplastic polymers, like polyethylene terephthalate glycol (PETG), exhibited differing resultant forces [21], emphasizing the necessity of comprehensive measurement during device assessment.

Regarding the force magnitudes, experimental investigations involving 24 patients and commercial devices unveiled notable findings. The application of force at the lingual base was studied using both a comparative Macintosh laryngoscope and a commercial Glidescope VLP. Data acquired through sensors, encompassing metrics such as peak, average, and impulse forces, revealed a substantial reduction exceeding 50% in the peak force (25 N) when utilizing the Glidescope video laryngoscope, as compared to the average force of 41 N exerted with the Macintosh laryngoscope metal during laryngoscopy [30].

In a comparative manner, the study conducted by Rassam in 2005 [23] entailed the integration of vertical force measurements through the employment of a mass balance (Mettler PM16, Mettler Instruments, High Wycombe, UK) and horizontal force measurements via a force transducer (AFG 500 N, Mecmesin Ltd, Horsham, UK). The amalgamation of these distinct forces culminated in a resultant force, whereby the pinnacle force denoted the utmost value during laryngoscopy. A comprehensive examination spanning over a thousand cases demonstrated a closely aligned peak force exhibited by the Macintosh laryngoscope metal (84N) [23], resembling the force output of PETG (100N) [21]. Conversely, scrutiny of 1009 mannequin laryngoscopies revealed a peak force (vertical-to-horizontal force ratio) oscillating between 32 and 39 N upon deployment of both metal and plastic Macintosh blades. The duration of intubation averaged 5.1 s and was discernibly influenced by blade dimensions and angulation, rather than the experience of the anesthetist [23]. It is noteworthy that these values substantially deviated from those of the ABS prototype (18.8N) [12, 13].

Comparative evaluation of 20 different disposable and non-disposable blade materials, excluding polymer filament material, indicated that laryngoscopy duration exhibited a direct correlation with increased force. Consequently, lighter devices facilitate visualization and reduce intubation time. Moreover, the utilization of polycarbonate blades could be repeated for up to 100 intubations without damage or fracture—a figure analogous to predictions for polymer filament blades [8, 23].

With regard to blade angulation, the video laryngoscope models produced demonstrated a faithful replication of the conventional Macintosh laryngoscope model, specifically utilizing the number 3 blade pattern that attaches to the handle. The interrelation between the blade's angulation and its attachment to the handle emerged as a pivotal factor in diminishing the risk of equipment fracture and enhancing oral cavity accessibility. These enhancements were particularly pronounced in the context of the Macintosh model, where blade angulation was meticulously optimized to ensure optimal laryngeal visualization [7, 8]. Certain studies have underscored the significance of this attribute in enhancing laryngeal visualization, albeit without explicitly delineating an ideal blade angulation. These studies assert that angles ranging from 45° to 60° facilitate superior visualization without necessitating cervical hyperextension, with a preference for hyper-angled blades.

In contrast, an investigation comparing Glidescope blades with varying angulations in 162 patients indicated that a 70° angle yielded reduced intubation time and higher success rates, rendering it more preferable than the 90° angle [1, 24]. These ranges of angulations recur in technological documents [13, 28, 31].

Several device attributes contribute to ease of use and glottic visualization. An optimal distance of 5.5 cm from the blade’s tip was identified as ideal for microcamera positioning, enabling effective laryngeal visualization [19]. The incorporation of the handle in 3D-printed models enhances the video laryngoscope's ergonomic profile, consequently facilitating smoother intubation [2]. Notably, investigations involving commercial devices revealed that microcamera illumination diminishes over repeated use and subsequent sterilization cycles. Despite variations in luminosity, these alterations did not significantly impair visualization [23]. Conversely, the absence of a smartphone support mechanism for displaying microcamera images was identified as a hindrance to effective visualization. The detachment of images due to their presentation on a separate device extended laryngeal visualization time and the duration required for successful intubation [1, 17, 18].

The validation of the video laryngoscope prototype encompassed assessments conducted on simulation mannequins and experienced professionals, underscoring the preliminary testing phase of the equipment. The utilization of mannequins was recommended to ascertain the basic functionality of the device. However, the new blades must undergo patient-based testing before reaching the consumer market. Comparative tests with conventional equipment, such as metal and plastic blades, are imperative to identify the optimal performance of the device [23, 32].

During simulations involving mannequins, a standardized scale consistently informed the studies. Key evaluation parameters encompassed intubation time, laryngeal visualization time, and intubation success rates within the context of 3D-printed models. Primary parameters, particularly intubation time and success rate, were deemed essential for evaluation. Intubation failure was defined as instances requiring 120 s or more for intubation within a maximum of 3 attempts [33]. Intubation time was measured from the device’s oral cavity entry to the insertion of the Oro-tracheal tube through the vocal fold. The utilization of the 3D-printed VLP yielded shorter. Intubation time was measured from the device’s oral cavity entry to the insertion of the Oro-tracheal tube through the vocal fold. The utilization of the 3D-printed VLP yielded shorter intubation times, with a minimum of 5.1 s and an average of 13.3 s [8]. This minimum time aligns with the shortest duration recorded in conventional studies, at 5 s, involving diverse Macintosh blades in both metal and plastic [23]. The highest intubation success rate achieved was 94.1% [24].

The video laryngoscope is considered a semi-critical medical device, as it comes into contact with non-intact skin or intact mucous membranes, making it susceptible to contamination by microorganisms such as bacteria, fungi, viruses, or prions. As a result, these devices require a level of disinfection that can be achieved with the use of chemical disinfectants when dealing with stainless steel or smooth plastic devices. Medical devices classified as semi-critical should undergo at least medium to high-level disinfection after cleaning. The Centers for Disease Control and Prevention (CDC) and the American Society of Anesthesiologists recommend cleaning and disinfection with chemical agents, and the FDA recommends a minimum of 10 min contact time, although autoclaving for disinfection is considered the ideal method. A strong recommendation is that both the blade and the handle be thoroughly cleaned and disinfected to reduce the risk of infection [33, 34].

In a study conducted in Spain, 38% of laryngoscope devices were cleaned and disinfected, with no national protocol in place. Esler et al. [35] described that in the United Kingdom, 60% of healthcare services do not use disinfection protocols for laryngoscopes.

The disinfection process with disinfectant products or physical thermos disinfection should comply with the health regulations of each country. To enable patient testing, the disinfection methodology is one of the key considerations when seeking approval from hospital ethics committees, as it is essential to prevent contamination of the medical equipment and reduce the risk of infections among patients. This underscores and justifies the need for elucidating the best and most efficient disinfection methodology for the 3D video laryngoscope [33, 34]

### Limitations and risk of bias

In this study, limitations may be present in the selection phase and complete reading of the articles, leading to selection bias. In addition, comparisons are limited owing to the heterogeneity between studies.

## Conclusions

The paramount feature of the video laryngoscope is its hyper-angled blade, ideally positioned within the range of 70° to 90° [1, 8]. The jaw traction force required for this type of blade should approximate 25 N, while the minimum force capable of inducing deformation should fall between 84 and 100 N. Validation tests performed on simulation mannequins enable the assessment of prototypes and emphasize primary evaluation parameters, namely intubation time and success rate. These parameters are influenced by the degree of blade angulation. Nonetheless, patient tests must precede product availability in the market, as they facilitate the validation of other parameters not encountered in simulation mannequins.

### Supplementary Information

**Additional file 1: Table S1. **Studies encountered according to the type of publication/study, authorship, title, journal/institution, country of study, and year of publication.

**Additional file 2: Table S2. **Description of technological documents.

## Data Availability

The data that directly support the study results are in Mendeley Reference Manager (https://www.mendeley.com/reference-manager/library/all-references), and technological database (https://worldwide.espacenet.com/) (https://patentscope.wipo.int/search/pt/advancedSearch.jsf) (https://busca.inpi.gov.br/pePI/jsp/patentes/PatenteSearchAvancado.jsp).
