# Correction: Advancements in additive manufacturing for video laryngoscopes: a comprehensive scoping and technological review

**DOI:** 10.1186/s13643-024-02453-z

**Published:** 2024-01-17

**Authors:** Ana Cristina Beitia Kraemer Moraes, Chiara das Dores do Nascimento, Everton Granemann Souza, Mauricio Beitia Kraemer, Mauricio Moraes, Neftali Lenin Villarreal Carreno, Evandro Piva, Rafael Guerra Lund

**Affiliations:** 1 Pelotas Dental School, Graduate Program in Dentistry, Federal University of Pelotas, Pelotas, RS 96010‑560 Brazil; 2 Master’s Degree in Electronic and Computer Engineering, Center for Social and Technological Sciences, Catholic University of Pelotas, Pelotas, RS 96015‑560 Brazil; 3 Faculty of Medicine, São Francisco University, Bragança Paulista, SP 12916‑900 Brazil; 4 Faculty of Medicine, Federal University of Pelotas, Pelotas, RS 96010‑560 Brazil; 5 Graduate Program in Materials Science and Engineering, Technological Development Center, Federal University of Pelotas, Pelotas, RS 96010‑610 Brazil

**Correction: Systematic Reviews 12:236 (2023)**

10.1186/s13643-023-02406-y

Following publication of the original article [1], the name of one of the authors, Dr. Neftali Lenin Villarreal Carreno, has been inadvertently published with a typographical error as “Neftali Lenin Villareal Carreno”. The original article has been corrected.
