# Supplementary material for: Advancements in additive manufacturing for video laryngoscopes: a comprehensive scoping and technological review
Source: Syst Rev. 2023 Dec 14;12:236. doi: 10.1186/s13643-023-02406-y (PMC10720237; doi:10.1186/s13643-023-02406-y)
Supplement: Supplementary file 1 — Additional file 1: Table S1. Studies encountered according to the type of publication/study, authorship, title, journal/institution, country of study, and year of publication. [file 13643_2023_2406_MOESM1_ESM.docx]

SUPPLEMENTAL TABLE 1. Studies encountered according to the type of publication/study, authorship, title, journal/institution, country of study, and year of publication.

| **Publication/Study Type** | **Author** | **Title** | **Journal** | **Country** | **Year** |
| --- | --- | --- | --- | --- | --- |
| Article/Observational Study | Paydafar J; Wu X; Halter R. | MRI- and CT-Compatible Polymer Laryngoscope | Otolaryngology-Head and Neck Surgery | USA | 2016 |
| Article/Observational Study | Christiansen J; Swanks S; Hsiung R. | 3D printing the affordable video laryngoscope | Anesthesia & Analgesia | USA | 2017 |
| Article/Controlled Randomized Clinical Trial | Cohen T; Nishioka H. | Comparison of a low-cost 3D printed video laryngo-borescope blade versus direct laryngoscope for simulated endotracheal intubations | Anesthesia & Analgesia | USA | 2017 |
| Article Cross-sectional Study | Cabrera D; Massano G; Fernández S; et al. | Low-cost laryngoscope video developed using 3D printing technology | Revista Chilena de Anestesiología/ Chilean Society of Anesthesiology | Argentina | 2018 |
| Article/Observational Study | Dinsmore M; Sin V; Matava C. | 3D Printed Thermal Laryngoscope | Journal of Medical Systems | Canada | 2019 |
| Abstract/Cross-sectional Study | Cardoso G.C; et al. | Use of 3D-printed video laryngoscope in IOT teaching during the 2019/2020  pandemic | 40th Scientific Week of the Clinical Hospital of Porto Alegre | Brazil | 2020 |
| Abstract/Case Study | Habib M; Sims R; Inziello | Design and Optimization of Patient -Specific Pediatric Laryngoscopes | Proceedings of the 2020 Design of Medical Devices Conference (DMD2020) | USA | 2020 |
| Article/Observational Study | Huysamen H ; Kinnear W ; Fonternel T et al. | 3D Printed Laryngoscope for Endotracheal Intubation | South African Journal of Industrial Engineering | South Africa | 2020 |
| Article/Cross-sectional Study and Randomized Clinical  Trial | Lambert C; John S; John A. | The ‘Tansen videolaryngoscope’: a low-cost device for resource-limited settings, combining a smartphone-compatible endoscope and three-dimensional printed blade. | European Journal of Anesthesiology | UK | 2020 |
| Article/Letter to the Editor | Almeida V; Almeida V; Campos G. | Challenges of prototyping, developing, and using video laryngoscopes produced by inhouse manufacturing on 3D printers | Brazilian Journal of Anesthesiology (English Edition) | Brazil | 2021 |
| Article/Observational Study | Quiroga J; Flor O; Solórzano S; et al. | Design of a Videolaryngoscope with sensor and pressure alert | Revista Athenea en Ciencias de la Ingeniería | Ecuador | 2021 |
| Article/Case Report Study | Maya Marcílio C; Meléndez Ordoñez J; Montes Ríos A | Hybrid laryngoscope: A reasonable option cost for approaching the airway difficulty. A case report | Revista Chilena de Anestesología Chilean Society of Anesthesiology | Mexico | 2021 |
| Article/Observational Study | García R.M.C | Low-cost video laryngoscopy with 3D printing technology. A concept proof | Anesthesia and Analgesia | Spain | 2021 |
| Article/Observational Study | Hughey et al, 2021 | 3D-printed laryngoscope for military austere environments | BMJ Military Health | USA | 2021 |
| Article/Controlled Randomized Clinical Trial | De Villiers C ; Alphonsus C ; Eave D ; et al. | Innovation in low-cost video-laryngoscopy: Incubator V1-Indirect compared with Storz C-MAC in a simulated difficult airway | Trends in Anesthesia and Critical Care | South Africa | 2021 |
| Article/Observational Study | Triantopoulos A; Triantopoulos O; Kostopoulos V; et al. | Presenting an innovative 3D-printed video laryngoscope | Trends in Anesthesia and Critical Care | Greece | 2021 |
| Article/ Randomized controlled cross-over study | Ataman A; Altina E. | Comparison of a commercial 3D fabricated laryngoscope (Airangel ® with a widely used video laryngoscope (Glidescope ®): Randomized controlled cross-over study | Trends in Anesthesia and Critical Care | Turkey | 2021 |
| Article/Systematic Review | Hamal PK, Yadav RK, Malla P | Performance of custom made videolaryngoscope for endotracheal intubation: A systematic review. | PLoS One | Nepal | 2022 |
|  |  |  |  |  |  |
| Article/Observational Study | Londoño MJ, Arango, JF, Izasa JF | Design and Development of a low-cost pediatric videolaryngoscope | Proceedings of the International Conference on Engineering Design (ICED23) | Bordeaux, France | 2023 |
| Article/Observational Study | Kienle LL, Schild LR, Bohm F, Grasslin R, Greve J, Hoffmann TK, Schuler PJ | A novel 3D-printed laryngoscope with integrated working channels for laryngeal surgery | Frontiers in Surgery | Germany | 2023 |
| Article/Randomized Cross-over Study | Fonternel T, Rooyen H, Joubert G, Turton E | Evaluating the usability of a 3D-printed Video Laryngoscope for tracheal Intubation of a Manikin | Medical Devices: Evidence and Research | South Africa | 2023 |
