# Supplementary material for: Advancements in additive manufacturing for video laryngoscopes: a comprehensive scoping and technological review
Source: Syst Rev. 2023 Dec 14;12:236. doi: 10.1186/s13643-023-02406-y (PMC10720237; doi:10.1186/s13643-023-02406-y)
Supplement: Supplementary file 2 — Additional file 2: Table S2. Description of technological documents. [file 13643_2023_2406_MOESM2_ESM.docx]

**SUPPLEMENTAL TABLE 2.**

Description of technological documents

| Publication/Year | Country | Title | Inventor | Claimed |
| --- | --- | --- | --- | --- |
| ES2524654A1·2014-12-10  PCT/ES2015/070013  WO2015104444A1 | Spain | Video-laryngoscope blade with connection to smartphones (Machine-translation by Google Translate, not legally binding) | CARNER BONET BERNART | Characterized Video-laryngoscope blade with connection to smartphones by comprising anatomical blade; a tunnel-guide of semirigid plastic, suitable for the passage of a boogie, which tunnel-guide runs along the underside of the blade. Comprising a chamber and a lighting element type LED; said system image capture being connected to wires inside the blade and connecting to a smartphone. |
| WO2019075588A1·2019-04-25  PCT/CL2018/050100  (Industrial applicability in few claims) | Chile | Medical device for endotracheal intubation of humans and production method thereof | JUDITH BORDONES CARTAGENA | Characterized because it comprises a handle, a folding shovel, a video image system, and a washing system that pass through the folding blade and a channel in the left side to slide an endotracheal tube. Where said parts are manufactured in a resistant material such as a metal of surgical use and / or a polymer for medical use and / or a composite material for medical use that resists compression and / or a combination among themselves. |
| CL2017002471U1·2018-02-02 | Chile | Clinical teaching video laryngoscope with a plastic structure of biodegradable plant origin, developed with 3D technology, which allows the coupling of a camera to visualize anatomical structures in human beings for the realization of endotracheal intubation. | MAX RODRIGO CORVALAN ASTUDILLO | The device its body manufactured in a single structure allows to make it portable.  No more claims are described in patent. |
| GB2575110A  WO2020003192A1·  2020-01-02  PCT IB2019/055449  (Industrial applicability in all claims) | United Kingdom | WIRELESS LARYNGOSCOPE | EAVE DYLAN; DE VILLIERS CHRISTIAAN TERTIUS; DE VILLIERS JACQUES ALBERT. | Characterized by the single part is made from injection-molded plastics. Wherein the insertion member is angled or curved from the first axis to the second axis through a total angle of between 100 and 135 degrees. Has uneven sides fouled, to thereby dissuade a user from cleaning the laryngoscope for reuse. A flat surface extending along a plane that is angled between 30 and 60 degrees from the first axis. |
| CN110724310A·2020-01-24 | China | Degradable material for anesthetic laryngoscopes, and preparation method thereof | WANG QIUPING | A degradable material for anesthesia laryngoscope, characterized in that it is made of raw materials of vinyl-modified polylactic acid, glucopyranoside, vinyl, PGA fiber, itaconic acid, hydroxyapatite, and glycan. |
| BR 20 2019 014222 2 U2 | Brazil | CONFIGURATION APPLIED IN LARYNGOBOROSCOPE OR VIDEO LARYNGOSCOPE 3D FOR SMARTPHONES. | IVAN DIAS FERNANDES PEREIRA / THIAGO MATTIA | Characterized by a blade in 3D impression using POLICARBONATO, ASA, ABS, PETG, TRYTAN, NYLON); A blade with insertion of the camera (borescope with connexon USB, 7mm and 6 LEDs) |
| GR20180100390A·2020-04-15 | Greece | VIDEO LARYNGOSCOPE FOR INTUBATION | TRIANTOPOULOS ORESTIS-KONSTANTINOS ALEXIOU; PAPANAOUM MAGDALINI EVANGELOU; KOSTOPOULOS VASILEIOS EVANGELOU; TRIANTOPOULOSALEXIOS GEORGIOU | Characterized by the fact that the tracheal tube is with a slope of 6.82 and 3.73 with respect to the axis. Adapted to the anatomical features of the operator and made of polymeric material with 3D printing technology and with 3 different front configurations. |
| BR 102020026194 0  21/12/2020 | Brazil | ANATOMIC VIDEOLARINGOSCOPE | ANA CRISTINA BEITIA KRAEMER MORAES / CHIARA DAS DORES DO NASCIMENTO / EVERTON GRANEMANN SOUZA | Characterized by two parts connected and take an internal channel with a micro camera into the blade, and symmetrical half and external channel to the passage of aspiration. The angle is specific to guarantee the safe in the access of the airway |
